# An association between multiculturalism and psychological distress

**DOI:** 10.1371/journal.pone.0208490

**Published:** 2018-12-06

**Authors:** Frank L. Samson

**Affiliations:** UCLA Institute for Research on Labor and Employment, Los Angeles, California, United States of America; University of Texas Medical Branch at Galveston, UNITED STATES

## Abstract

Amidst increasing focus on rising rates of substance abuse and suicide among white Americans and extending prior research on intergroup attitudes and health, this study examines a novel factor associated with psychological distress: disagreement with multiculturalism. Using the Portraits of American Life Study (N = 2,292), logistic regressions indicate that for Whites and Hispanics, increased likelihood of psychological distress (depression, hopelessness and worthlessness) is associated with stronger disagreement with multiculturalism, measured as “If we want to create a society where people get along, we must recognize that each ethnic group has the right to maintain its own unique traditions.” For Blacks, however, attitudes toward multiculturalism are not associated with psychological distress. Future research might determine if these results can be replicated, and if so, identify the causal mechanism(s) at work.

## Introduction

Recent studies have identified an increasing trend in death rates among white Americans [1–4]. These deaths have often been tied to substance abuse and suicide, or “deaths of despair” [2, 4]. Researchers have described this rise in death rates among white Americans as akin to an “epidemic of hopelessness” [5] or an “epidemic of despair” [4], while Nobel Prize-winning economist Angus Deaton has stated, “It’s a loss of hope” [6]. Many of the proposed explanations have focused on economic factors such as income, labor market outcomes, globalization, etc., with some gestures towards psychological concepts, such as (failed) expectations for attaining significant life events [2]. While researchers have identified this epidemic of despair disproportionately among less educated middle-aged white Americans [2], SAMHSA (Substance Abuse and Mental Health Services Administration) reports that treatment admissions for both heroin and non-heroin opiates are highest among younger whites in their 20s and 30s [7]. Other researchers report that heroin use over the past 50 years has “migrated… to more affluent suburban and rural areas with primarily white populations” [8]. Not to be limited to rural and suburban areas, researchers note that the epidemic is urban as well [2]. White-majority spaces have even been tied to an increase among non-white populations in the risk of non-medical prescription painkiller misuse; longitudinal data revealed that black youth who attended majority-white schools were at higher risk a decade later of non-medical prescription painkiller misuse than black students who attended majority black schools [9]. Considering the substantial time and resources devoted to researching these issues, which afflict young and old, under-resourced and affluent, it is quite striking that only one primary commonality stands out in the current crisis: whiteness. This study’s central contribution is to propose an additional factor that could be associated with apparently race-related despair or psychological distress, an additional differentiating factor beyond simply whether someone is a white American. This study looks to emerging research on intergroup attitudes and mortality to propose this additional factor, one that may cut across socioeconomic and demographic categories.

Public health researchers have recently begun to more fully reveal associations between intergroup attitudes and mortality. Anti-gay prejudice has been linked to increases in cardiovascular-related death [10], while racial prejudice is associated with all-cause mortality [11]. Furthermore, implicit racial bias (implicit association test) and explicit bias (feelings thermometer difference scores on coldness/warmth of blacks compared to European Americans) both predicted later circulatory-related (e.g. heart disease) mortality. While these studies have largely identified effects on the cardiovascular system, researchers have yet to test whether an association might also exist between intergroup attitudes and the psychopathological symptoms at the center of the aforementioned rise in death rates, symptoms of psychological distress such as hopelessness, worthlessness, and depressed mood.

A variety of social psychological studies examining changing demographics and diversity point to the possibility of such an association. Exposed to an experimental cue indicating that ethnic minorities are expected to become a population majority, white study participants expressed increases in racial bias and decreases in warmth towards minorities [12]. In another study, exposure to an experimental cue depicting white minority status in the future elicited increased feelings of both anger and fear towards ethnic minorities [13]. Aside from changes in population demographics, simply presenting an organization as endorsing pro-diversity policies was enough to experimentally elicit physiological markers indicative of threat (i.e. increases in total peripheral resistance signaling vasoconstriction) among white male study participants [14], another finding consistent with the aforementioned studies identifying cardiovascular consequences. For the purposes of the present investigation, what is perhaps noteworthy about the second study–diversity-related experimental elicitations of anger and fear–is that it suggests a plausible pathophysiological pathway for the present study. Both emotions are not only tied to cardiovascular outcomes, likely mediated through endocrine release via the hypothalamus-pituitary-adrenal axis, but they also suggest possible involvement of specific cognitive and emotionally-relevant regions and neural networks in the brain, the organ most proximal to psychopathology and psychological distress.

It is important to examine how growing diversity, and possible challenges adapting to diversity, may relate to psychological distress. One possible response to growing diversity is the celebration of diversity, a position referred to frequently as either cultural pluralism [15] or multiculturalism [16]. An alternative response to growing diversity is the rejection of the differences such diversity may represent, a model of ethnoracial relations described as assimilation [15]. In at least two studies of white participants, indicators of both ethnic outgroup threat and loss of majority status among whites was associated with an increase in endorsement of cultural assimilation and a decrease in diversity endorsement [17]. The tension between multiculturalism and assimilation has long been a weighty political issue in the United States [15]. While one-way assimilation has historically been the primary approach to immigrant incorporation in the United States [18], multiculturalism’s more recent rise to ascendancy can be traced to protest movements in the 1950s and 1960s [19].

The present study bridges these literatures on intergroup attitudes, diversity, and health outcomes with the concern over “despair” stated at the outset. Given the popularity enjoyed by multiculturalism in the United States today, the present paper examines: is disagreement with multiculturalism associated with psychological distress? Unlike many of the studies on intergroup attitudes and health reviewed earlier, because the data examined in the present study is cross-sectional, the present inquiry will not propose or test any causal mechanism linking intergroup attitudes and psychological distress. Rather, the present study sets out to more modestly identify an attitudinal association by testing the following hypothesis: *disagreement with multiculturalism is associated with a higher likelihood of psychological distress*. While much of the discussion on “deaths of despair” has focused on white Americans, the present study will also test this hypothesis among minority populations. Until the more recent shift towards addressing health disparities and health equity, minority health has been relatively neglected in medical and health research studies in the U.S., so it is crucial to include minority populations in any exploration of potentially novel health-associated factors [20, 21].

## Methods

### Sample

To explore the relationship between multiculturalism and psychological distress, this study draws upon the Portraits of American Life Study [22]. The Portraits of American Life Study (PALS) is a panel study, consisting of adult respondents (18 years of age or older) across the United States. Data for the first wave were collected during April to October of 2006 from a random sample constructed using a five-stage design with oversampling of black and Hispanic respondents. Interviewers conducted face-to-face interviews in English, Spanish, or both, using a paper-and-pencil instrument for general questions and audio computer-assisted self-interviewing for items pertaining to sensitive topics (e.g. moral attitudes, attitudes about race and ethnicity, deviance, etc.). Respondents were paid $50 for completing the interview, which averaged 80 minutes. The response rate was 58% [22].

The final PALS sample consisted of 2,610 respondents, with 1,263 White respondents, 528 African Americans, 520 Hispanics, 177 Asians, 26 Native Americans and Pacific Islanders, 60 who identified as mixed race, and 36 respondents who identified as Other. Due to the small sample sizes of ethnoracial groups outside the three largest ethnoracial groups, only White, Black, and Hispanic respondents are analyzed. The valid N for this study after multiple imputations to recover missing data was 2,292 respondents (or 99% of the White, Black, and Hispanic samples).

### Measures and analyses

Psychological distress is constructed by identifying respondents who responded yes to all of three mood disorder symptoms: depression, hopelessness and worthlessness. Each is measured with a yes/no response using the following items:

- In the past 12 months, have you ever had two weeks or longer when nearly every day you felt sad, empty, or depressed for most of the day?
- In the past 12 months, have you ever had two weeks or longer when nearly every day you felt hopeless?
- In the past 12 months, have you ever had two weeks or longer when nearly every day you felt worthless?

Prior research has used feeling worthless and hopeless as part of multiple-item scales measuring nonspecific psychological distress; the time designated in these items are typical measures of episodic major depressive disorder [23]. It is worth noting that hopelessness and worthlessness (low self-esteem) are among two additional symptoms, concurrent with depressed mood, necessary to meet the DSM V diagnostic criteria for persistent depressive disorder (previously dysthymia), though a longer time course (2 years for adults, 1 year for adolescents or children) is required [24]. In light of these considerations, this study’s binary outcome variable is constructed in a two-step process. First, responses to the three items were summed to verify internal reliability (alpha = 0.81). Second, respondents who score a 3, or answering yes to all three items, were recoded as 1 on a binary outcome variable, with 0 representing those below a potentially diagnostic threshold for dysthymia if symptoms persisted long term.

The following survey item captured agreement with multiculturalism: “If we want to create a society where people get along, we must recognize that each ethnic group has the right to maintain its own unique traditions.” This item was coded strongly disagree at the low end of a five-point scale with strongly agree coded at the top, and somewhat disagree, neither agree nor disagree, somewhat agree in between. The item was reverse-coded to facilitate ease of interpretation based on the hypothesis regarding multiculturalism *disagreement*. A slight variant of this item has been used previously as part of a multi-item multiculturalism scale in prior social psychology and personality research [25]. The original variant—If we want to help create a harmonious society, we must recognize that each ethnic group has the right to maintain its own unique traditions–was also asked on the General Social Survey (GSS) in 2002, the GSS being one of the three “gold-standard” public opinion surveys in the United States [26]. While multiple item measures are preferred, prior studies have used similar single item multiculturalism measures [27, 28], and a plethora of studies are now emerging supporting the utility of single-item measures in social psychology and personality research [29, 30].

This study controlled for various factors in the estimation models (see Appendix A for question wording). The significant associations between racial attitudes and individual factors such as age, education, income, gender, and political ideology have already been well established [31]. Likewise, age [32], education [33], income [32], and gender [32] have been tied to dysthymia, while political ideology, though not specifically tied to dysthymia, has been associated with affective mood [34]. Age was controlled as a continuous variable, while education involved recoding an ordered, 12-category item capturing highest degree completed into five binary variables: less than high school, high school or GED (reference category), some college (including vocational degrees, associates degrees, and two year religious degrees), bachelor degree, and postgraduate degrees. Household income was constructed by coding the midpoint of a 19-category ordinal scale, and dividing by 10,000; multiple imputations were used to deal with missing data. Gender and nativity were coded as dichotomous variables with 1 indicating female and foreign-born, respectively. While fewer studies have identified associations between racial attitudes and nativity [35], at least compared to the other individual characteristics listed above, nativity has also been tied to differences in both dysthymia and depression [36]. Political ideology was controlled using two dummy variables indicating conservative and middle of the road political ideology, relative to liberals and those who haven’t thought much about political views or activities as omitted categories. Stress was controlled using a summation of 12 items (alpha = 0.65) to construct a scale for stressful life events in the past 3 years (e.g. serious illness, family death, marital separation, police problems, etc.). It should be noted that the stressful life events scale also contains three items indicating economic vulnerability (unemployed, fired, and financial crisis), an important factor in research on “deaths of despair” [2]. Controlling for this measure of stress would also partially account for economic vulnerability as a confounding factor.

Finally, the models controlled for social psychological factors already studied in the health literature. One item asked if the respondent had been treated unfairly in the past 3 years because of their race, to control for perceived discrimination [37]. An individual who has experienced racial discrimination may be both more likely to experience psychological distress and, at least according to Huntington [38, 43], may be less tolerant of cultural differences (lower on multiculturalism agreement). In light of prior studies depicting an association between attachments to one’s racial or ethnic group and mental health, the models included a control variable measuring respondents’ feelings of closeness to their racial group [39]. An individual who felt close to their racial group may be both more accepting of ethnic differences (higher on multiculturalism agreement) while enjoying the salubrious mental health effects of closeness to their racial group. Racial group closeness was included as a four-category ordinal variable, encompassing responses spanning “not at all,” “somewhat,” “very,” and “extremely.” Higher values on this scale indicated feelings of greater close connection to one’s racial group.

### Modeling strategy

This study uses binary logistic regression to examine the relationship between multiculturalism and psychological distress, adjusted for various socio-demographic characteristics and net of various controls. As with household income, multiple imputation with chained equations was used to address missing data [40], though imputed values on the dependent variable were not utilized in the analyses. Missing data were imputed on multiculturalism (1.9%), income (10.3%), political ideology (0.7%), perceived discrimination (0.2%), and feelings of racial closeness (1.4%). The main results did not differ between models estimated with imputed missing data compared to models estimated with only complete cases. Following convention for studies exploring the health of ethnoracial groups, and due to recent research documenting the complexity involved in interpreting interaction effects in nonlinear models [41], this study estimates separate models for each of the ethnoracial group samples alone rather than employing race-based interaction terms in a pooled sample. Race/ethnicity was assessed in the survey by asking: What race or ethnic group do you consider yourself? (That is, are you white, black, Hispanic, Asian American, Pacific Islander, American Indian or of mixed race?)”

## Results

Table 1 describes the weighted sample along a few key dimensions. About 10% of the sample experienced two-week or longer bouts of all three psychological distress indicators at some point in the past twelve months. Though not listed in Table 1, 70% reported no distress, 13.9% reported one distress indicator, and 5.6% reported two indicators. Notably, seventy percent expressed at least some agreement with the tenets of multiculturalism, which is suggestive of its ascendant status. S1 Appendix presents the psychological distress outcome distributions across categorical explanatory variables.

**Table 1:** Sample distributions of variables by race subgroup.

	Full sample	Whites	Blacks	Hispanics
Observations (N = 2,292)		75.2%	11.6%	13.3%
Psychological Distress				
Less than three indicators	89.5%	90.0%	88.7%	87.5%
All three indicators (depressed,	10.5%	10.0%	11.3%	12.5%
hopeless, and worthless)				
Multiculturalism Agreement				
Strongly disagree	6.9%	6.9%	8.6%	7.1%
Somewhat disagree	6.9%	7.8%	5.8%	4.1%
Neither	14.4%	14.3%	14.9%	17.4%
Somewhat agree	29.8%	32.8%	24.3%	20.9%
Strongly agree	40.1%	38.2%	46.4%	50.4%
Missing	1.9%			
Age (years, mean)	45.4	46.9	42.7	39.3
Highest Degree Completed				
Less than high school	12.2%	8.1%	16.3%	31.3%
High school or GED	41.6%	40.7%	48.2%	40.9%
Vocational, Associates, or Two Year Religious	18.3%	18.8%	19.3%	14.6%
Bachelors	16.0%	18.5%	9.3%	7.8%
Masters, Doctorate, Professional, Other	11.8%	13.7%	6.8%	5.4%
Household income	$53,150	$64,554	$39,947	$42,039
Gender				
Male	48.2%	48.3%	44.0%	50.9%
Female	51.8%	51.7%	56.0%	49.1%
Nativity status				
U.S.-born	89.0%	96.6%	93.1%	42.3%
Foreign-born	11.0%	3.4%	6.9%	57.7%
Political Ideology				
Conservative	28.7%	31.8%	17.9%	21.4%
Middle of the Road	24.7%	27.6%	14.8%	18.0%
Liberal	20.4%	20.0%	23.6%	21.1%
Haven't Thought	25.5%	20.5%	43.5%	39.4%
Missing	0.7%			
Treated unfairly because of race in past 3 years				
No	87.4%	92.4%	65.5%	80.1%
Yes	12.3%	7.6%	34.5%	19.9%
Missing	0.3%			
Feel closely connected to racial group				
Extremely	19.8%	16.2%	33.9%	30.0%
Very	32.7%	31.3%	41.4%	37.1%
Somewhat	35.6%	40.6%	22.0%	23.6%
Not at all	10.4%	11.9%	2.7%	9.3%
Missing	1.4%			
Stressful life events (mean)	2.52	2.49	3.03	2.23

Data: Portraits of American Life Study (2006)

Table 2 contains the multivariate results. Providing support for the hypothesis, the likelihood of psychological distress is higher as disagreement with multiculturalism increases among White respondents (b = 0.30, p<0.01, two-tailed) and among Hispanic respondents (b = 0.29, p<0.05, two-tailed). Unexpectedly, for Black respondents, multiculturalism is not significantly related to psychological distress.

**Table 2:** Logit models estimating likelihood of psychological distress, by multiculturalism and controls.

	Whites		Blacks		Hispanics	
	N = 1,254		N = 524		N = 514	
	b	SE	b	SE	b	SE
**Multiculturalism Disagreement**	0.30**	(0.11)	0.07	(0.11)	0.29*	(0.14)
Age (in years)	0.003	(0.007)	-0.009	(0.01)	0.01	(0.01)
Highest Degree Completed^a^						
Less than high school	0.35	(0.37)	1.36*	(0.54)	0.35	(0.37)
Vocational, Associates, or Two Year Religious	-0.55+	(0.31)	-0.18	(0.61)	-1.01	(0.68)
Bachelor's degree	-0.07	(0.48)	-0.83	(0.60)	-0.004	(0.52)
Masters, Doctorate, Professional, Other	-0.73	(0.45)	-0.50	(0.86)	-1.30	(1.09)
Household income (tens of thousands)	-0.10**	(0.03)	0.01	(0.08)	-0.01	(0.07)
Female	0.56*	(0.27)	-0.50	(0.49)	1.04**	(0.36)
Foreign-born	0.18	(1.04)	-0.70	(0.92)	-0.65**	(0.22)
Political Ideology						
Conservative	-1.07**	(0.32)	0.37	(0.54)	-0.09	(0.52)
Middle of the road	-0.71**	(0.22)	0.89+	(0.53)	0.11	(0.52)
Treated unfairly because of race in past 3 years	-0.29	(0.45)	0.49+	(0.33)	0.03	(0.41)
Feel closely connected to racial group	-0.32*	(0.14)	-0.12	(0.24)	-0.32+	(0.18)
Stressful life events	0.54***	(0.07)	0.26***	(0.07)	0.61***	(0.09)
F-statistic	13.5***		8.5***		17.3***	

Data: Portraits of American Life Study (2006)

Notes: Standard errors in parentheses

*** p<0.001.

** p<0.01.

* p<0.05.

+ p<0.10 (two-tailed).

Omitted Categories: ^a^High school/GED

**Fig 1:**
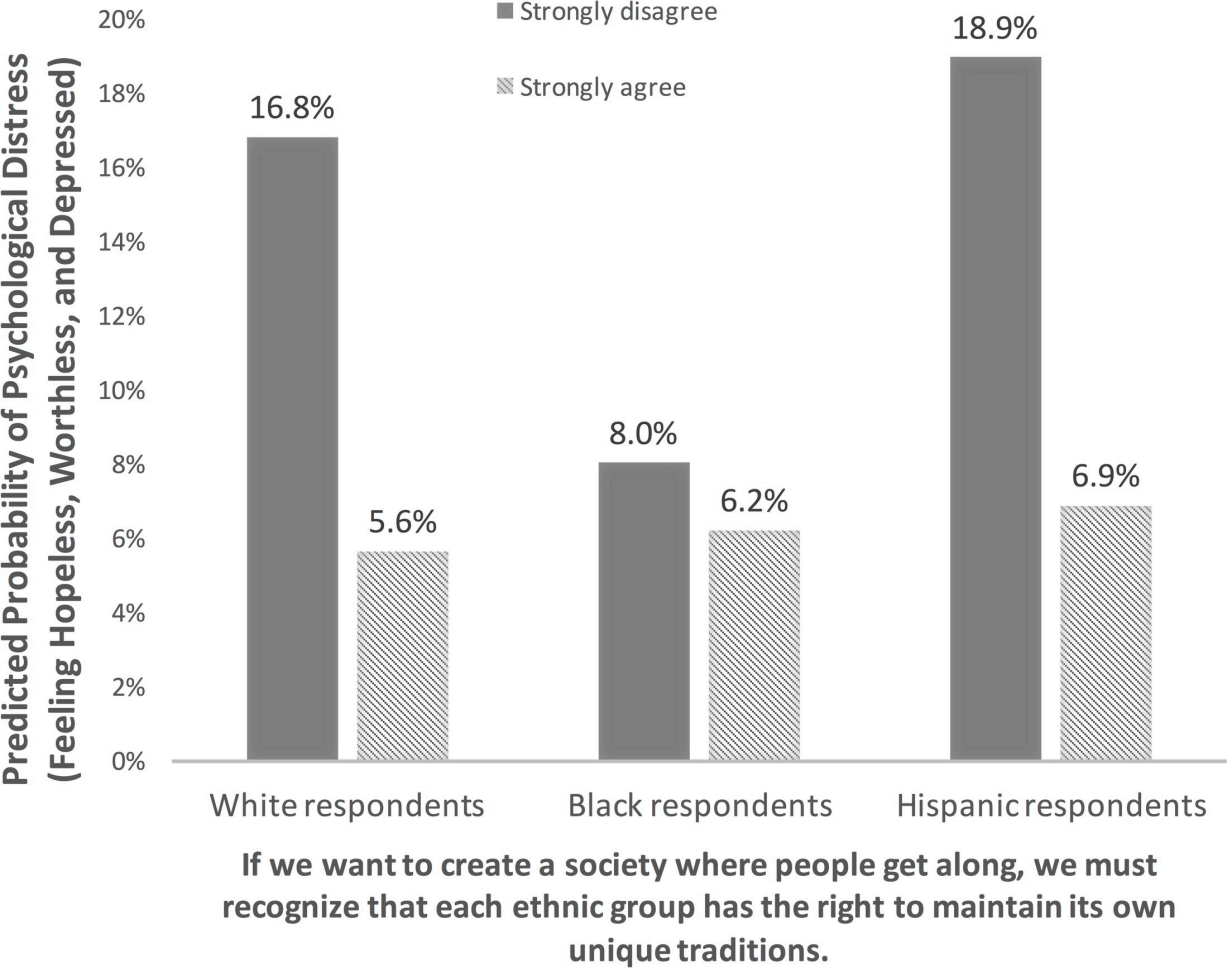
Predicted probability of psychological distress by multiculturalism agreement.

To more clearly illustrate the association between multiculturalism attitudes and psychological distress, Fig 1 presents predicted probabilities of psychological distress for a simulated respondent. The coefficients in Table 2 were applied to a respondent of average age, income, feelings of racial closeness and general stress level, who was also a high school graduate, male, U.S. born, politically liberal, and had not been treated unfairly because of race in the preceding three years.

The difference in predicted probabilities between those who strongly agree and strongly disagree with multiculturalism is striking. White respondents who strongly agreed with multiculturalism had a 5.6% probability of feeling psychological distress, while a Hispanic with similar views had a 6.9% probability of feeling psychological distress for two weeks or longer. In marked contrast, a white respondent who strongly disagreed with multiculturalism had a 16.8% probability of feeling psychological distress for two weeks or longer in the past year, while a Hispanic respondent who also strongly disagreed had a 19% probability.

## Discussion

The data analyses partially supported the hypothesis proposing an association between multiculturalism and psychological distress among whites and Hispanics. Notably, recent research on the “epidemic of despair” also found that at least among young adults, the highest increase in deaths from suicide between 1999–2001 and 2013–2015 was found among whites and Hispanics [4]. To the extent that multiculturalism enjoys an ascendant position in U.S. society today, these results support prior health research that considers “the harmful effects of incongruence with the norms and values of the community majority” ([42]: 824).

The results also confirm findings from previous studies on race and mental health outcomes. That foreign-born Hispanic respondents had lower likelihoods of psychological distress confirms some findings looking at the “immigrant paradox” in mental health [43]. Perceived discrimination was positively related to psychological distress across all groups, reaffirming the link between perceived discrimination and depressive symptoms [44]. Finally, closeness to one’s racial group was also related to a decreased likelihood of psychological distress, for white respondents and marginally for Hispanic respondents, partially confirming prior research on how ethnic identity protects mental health [39]. The protective effect of ethnic identity among whites may surprise those most familiar with its relevance to minority groups. The effect may be an artifact of the diagnostic threshold coding for the outcome variable or perhaps related to whites increasing belief that anti-white bias is a bigger problem than anti-black bias [45]. Similarly, the absence of a significant and positive female association among the black sample might also be tied to outcome coding. Other results from the models seem to validate the sample, such as the higher risk of psychological distress among blacks with less than a high school education.

Prior research can shed light on the multiculturalism disagreement effect among Hispanics. Health researchers have linked minority mental illness to acculturative stress [46, 47]. Moreover, research has pointed towards important mental health differences among subgroups (e.g. Mexican, Puerto Rican, etc.) within the Hispanic population [43, 48]. To the extent that Hispanic ethnic groups with closer or longer ties with the United States may 1) believe more strongly in assimilation, but 2) psychologically come to terms with blocked mobility for many non-whites in the United States [49], perhaps leading to psychological distress, a supplementary analysis was conducted to control for some Hispanic subgroup (Mexican, Puerto Rican, and Central American) variation. None of these three subgroup indicators yielded a significant finding on the pre-dysthymic psychological distress outcome, and none had any notable influence on the main multiculturalism disagreement effect (results available upon request). It should be noted, however, that the PALS survey design was not intended, and therefore not optimized, to study Hispanic inter-group differences; a larger sample of Hispanics may yet yield statistically significant findings.

Some might argue that skin color (and racial) variation within Hispanic groups (e.g. brown or black Hispanics) could account for the association between multiculturalism and psychological distress. To the extent that brown and black Hispanics have experienced prejudice and have placed less emphasis on assimilating into a host society with racial discriminatory barriers, such an experience may be tied to lower levels of psychological distress. White Hispanics, on the other hand, may have greater faith in assimilation, and when confronted with host society discrimination, present with higher levels of psychological distress. To test these possibilities, ancillary analyses were conducted using the following follow-up question for respondents who identify as Hispanic: Do you consider yourself white, black, Asian, American Indian, or something else?” Among the Hispanic respondents in the unweighted sample, 2% identified as black Hispanic, 42% as white Hispanic, 3% as American Indian Hispanic, and 52% as other Hispanic. Introducing two dummy-coded variables in separate models–first, one for black Hispanics; then, one for black Hispanics and other Hispanics combined–did not yield significant results for the skin color variables nor did they change the main association between multiculturalism disagreement and psychological distress among Hispanics (results available upon request).

There are historical explanations that can account for the null finding among black respondents. Historically, blacks have been largely absent from discussions about assimilation, or its earlier term of “Americanization” [50, 51]. Because of this history, tensions between multiculturalism and assimilation might be less salient or consequential for the mental health of African Americans than for their white and Hispanic counterparts. Researchers have also found that perceptions of zero-sum conflict with Latinos and expressions of modern prejudice (racial resentment) are significantly associated with culture-related immigration attitudes (i.e. whether English should be the official language or whether one supports English-only ballots) for white but not for black respondents [52]. Future research might examine these theoretical possibilities in greater detail.

Among the present study’s timely contributions is the identification of an associated factor outside the scope of the economic focus of researchers studying the current rise in deaths of despair among white Americans [1, 2]. Researchers have mainly emphasized declining labor market opportunities and income, globalization, and technological changes as primary engines of the rise in these deaths [2]. The present study’s models control for socioeconomic status (e.g. income and education), recent job firing, recent unemployment status, and recent financial crisis–the latter three as items in the stress scale–all of which signal individual vulnerability to financial strain. Net of these socioeconomic and labor market factors, the multiculturalism attitude variable remains linked to psychological distress. Notably, researchers have difficulty using an income-centered narrative to explain why blacks have comparatively been spared from deaths of despair, instead citing “historian Carol Anderson [who] argued in an interview … that for whites ‘if you’ve always been privileged, equality begins to look like oppression’ ([2]: 29). While the data employed by economists were unable to speak to this alternative explanation, the present study’s data is better suited to do so. This study’s multiculturalism variable captures an “equality” of respect for unique ethnic traditions, which if perceived as a form of cultural “oppression” by some whites, may be associated with psychological distress. While speculative, as this interpretation represents a causal process beyond what the cross-sectional data allow, this study’s data do permit some advancement over the economic data that researchers have brought to the study of white American deaths of despair. This study’s results provide evidence identifying an associated factor that corroborates what economics-centered researchers have speculated as a possible alternative account but do not have the data to address.

The present study’s results also speak to contemporary academic and popular debates about a potential association between psychiatric illness and forms of cultural intolerance, such as racism [53–55]. While the American Psychiatric Association does not include racism in the Diagnostic and Statistical Manual of Mental Disorders [56], some psychiatrists have argued that racism should be considered a treatable psychiatric illness [57, 58]. Outside academia, television and social media commentators have also contentiously weighed in on a possible link between mental illness and racist statements and actions [59]. Law enforcement officials and the criminal justice system have also referred suspects to psychiatric evaluation after racist criminal acts [60, 61]. Dylann Roof, convicted of killing several African American worshippers at a Charleston, South Carolina church, admitted to being deeply depressed months before the June 2015 murders [61]. While proponents and opponents of an illness-racism association alike cite the stark paucity of research to bring to the debate, the present study provides some data to add to the conversation.

This study has some limitations that should prompt future research. First, the survey data, collected in 2006, might appear to be somewhat dated. However, not only is it one of the few nationally representative datasets to contain both health outcomes and intergroup attitudes, the year 2006 is also in the middle of the rising trend of white American death rates identified by researchers who have studied the phenomenon using mortality data from 1999 to 2015 [4]. Second, the primary independent variable and other key concepts relied upon single item measures. Future studies should employ multiple item measures for multiculturalism [62, 63], perceived discrimination [64–68], and a more expansive multiple-item measure of psychological distress [23]. Some may view the use of a single item multiculturalism measure as an insurmountable concern. However, with U.S. life expectancy having decreased for the first time in almost a quarter century [69], the urgency of a rise in death rates among whites, attributed in part to suicide and opiate abuse [70], conceivably outweighs the possible shortcomings of a minor measurement limitation. Again, that this perceived measurement limitation may not be ruinous is particularly highlighted by already published research that have used single-item measures of multiculturalism [27, 28], and other studies recognizing the utility of single-item measures [29, 30].

Lastly, there are the well-known limitations of working with cross-sectional data. This limits the present analysis to identifying multiculturalism disagreement as an associated factor rather than inferring causation. Notably, a causal argument whereby psychological distress leads to disagreement with multiculturalism advances a controversial claim, as it posits that disagreement with multiculturalism may be partially rooted in a psychiatric disorder. Alternatively, one might argue that third variables may account for both psychological distress and disagreement with multiculturalism. Future research should investigate possible sources of omitted variable bias.

In sum, the present study extends prior research examining the health consequences of social psychological concepts such as prejudice, intergroup bias, and intergroup affect [10, 11, 71, 72], to propose the possibility of a social psychological factor useful for those studying the recent rise in “deaths of despair” among white Americans [1, 2] and others studying psychological distress among Hispanics. A notable social psychological contribution is that multiculturalism disagreement as a factor associated with (pre-dysthymia) psychological distress is not diminished by controlling for life stress events such as unemployment and financial crisis, given the current research focus on economic rationales as fundamental drivers of the unexpected rise in white mortality. Future research might further investigate whether causal links may exist between strongly-held intergroup attitudes and psychological distress or psychopathology.

## Appendix A: Survey items

**Psychological Distress** (alpha = 0.81)

- In the past 12 months, have you ever had two weeks or longer when nearly every day you felt sad, empty, or depressed for most of the day? (Yes/No)
- In the past 12 months, have you ever had two weeks or longer when nearly every day you felt hopeless? (Yes/No)
- In the past 12 months, have you ever had two weeks or longer when nearly every day you felt worthless? (Yes/No)

**Multiculturalism** Attitude: If we want to create a society where people get along, we must recognize that each ethnic group has the right to maintain its own unique traditions (strongly agree, somewhat agree, neutral, somewhat disagree, strongly disagree).

**Age**: How old was he or she on his or her last birthday? (Household roster intake, including respondent)

**Education**: What is the highest level of schooling you have completed, or what is the highest degree that you have earned?

**Income**: Please tell me the number on the card that is your best estimate of your total household income in 2005.

**Race**: What race or ethnic group do you consider yourself? (That is, are you white, black, Hispanic, Asian American, Pacific Islander, American Indian, or of mixed race?)

**Female:** Is this person a male or a female? (Household roster intake, including respondent)

**Foreign-born**: Were you born in the United States?

**Political ideology**: These next several questions are about your political views and activities. When it comes to politics, do you usually think of yourself as. . . . very liberal, somewhat liberal, middle of the road, somewhat conservative, very conservative or haven’t you thought much about this?

**Perceived discrimination**: Can you think of any occasion in the past three years, that you felt you were treated unfairly because of your race?

**Racial group closeness**: How closely connected do you feel to your racial group (extremely close, very close, somewhat close, not close at all)?

**Life event stress** (alpha = 0.65)

Now please think about the past 3 years. In the past 3 years, have any of the following life events or problems happened to you …

1. … you yourself suffered a serious illness, injury, or an assault?
2. …a serious illness, injury, or assault happened to a close relative or spouse/partner.
3. … your parent, child, or spouse died.
4. … a close family friend or another relative (aunt, cousin, grandparent) died
5. .. .. you had a separation due to marital difficulties.
6. … you broke off a steady, romantic relationship.
7. … you had a serious problem with a close friend, neighbor, or relative.
8. … you had a serious problem with someone in your congregation.
9. … you became unemployed or you were seeking work unsuccessfully for more than one month.
10. … you were fired from your job.
11. … you had a major financial crisis.
12. … you had problems with the police or you had to make a court appearance.

## Supporting information

S1 Appendix Weighted distributions of psychological distress by categorical explanatory variables. (DOCX)
