# Supplementary material for: An association between multiculturalism and psychological distress
Source: PLoS One. 2018 Dec 6;13(12):e0208490. doi: 10.1371/journal.pone.0208490 (PMC6283566; doi:10.1371/journal.pone.0208490)
Supplement: S1 Appendix — (DOCX) [file pone.0208490.s001.docx]

**S1 Appendix. Weighted Distributions of Psychological Distress by Categorical Explanatory Variables**

|  | Psychological Distress (All 3 items) | | |  |  |
| --- | --- | --- | --- | --- | --- |
|  | | No | Yes | |  |
| Multiculturalism Agreement | |  |  | |  |
| Strongly disagree | | 81.0% | 19.0% | |  |
| Somewhat disagree | | 91.1% | 8.9% | |  |
| Neither | | 88.9% | 11.2% | |  |
| Somewhat agree | | 90.2% | 9.8% | |  |
| Strongly agree | | 90.9% | 9.1% | |  |
|  | |  |  | |  |
| Education | |  |  | |  |
| Less than high school | | 80.0% | 20.0% | |  |
| High school or GED | | 86.8% | 13.2% | |  |
| Vocational, Associates, or Two Year Religious | | 92.2% | 7.8% | |  |
| Bachelors | | 93.0% | 7.0% | |  |
| Masters, Doctorate, Professional, Other | | 95.7% | 4.3% | |  |
|  | |  |  | |  |
| Gender | |  |  | |  |
| Male | | 91.6% | 8.4% | |  |
| Female | | 87.0% | 13.0% | |  |
|  | |  |  | |  |
| Foreign-born | |  |  | |  |
| No | | 89.0% | 11.0% | |  |
| Yes | | 91.2% | 8.8% | |  |
|  | |  |  | |  |
| Political Ideology | |  |  | |  |
| Conservative | | 94.1% | 5.9% | |  |
| Middle of the Road | | 90.3% | 9.7% | |  |
| Liberal | | 89.4% | 10.6% | |  |
| Haven't Thought | | 82.5% | 17.5% | |  |
|  | |  |  | |  |
| Treated unfairly because of race in past 3 years | |  |  | |  |
| No | | 90.1% | 9.9% | |  |
| Yes | | 83.8% | 16.2% | |  |
|  | |  |  | |  |
| Feel closely connected to racial group | |  |  | |  |
| Extremely | | 90.1% | 9.9% | |  |
| Very | | 91.2% | 8.8% | |  |
| Somewhat | | 89.1% | 10.9% | |  |
| Not at all | | 83.1% | 17.0% | |  |

Data: Portraits of American Life Study (2006)
